# A One Welfare approach to identify socioeconomic vulnerability in families during investigations into companion animal abuse in Pinhais, Brazil

**DOI:** 10.1017/awf.2023.18

**Published:** 2023-03-02

**Authors:** Stefany Monsalve, Janaina Hammerschmidt, Micheli Ribeiro, Maria Vitoria Duarte Caleme, Solange Marconcin, Gizeli Filius, Rita de Cassia Maria Garcia

**Affiliations:** 1 Programa de Pós-graduação em Ciências Veterinárias, Universidade Federal do Paraná, Rua dos Funcionários 1540, Curitiba, Paraná, Brazil; 2 Especialización en Bienestar Animal y Etología. Fundación Universitaria Agraria de Colombia, Calle 170 No 54A-10, Bogotá, Colombia; 3 Divisão de Bem-Estar Animal, Secretaria Municipal de Meio Ambiente, Prefeitura de Pinhais, Rodovia Deputado João Leopoldo Jacomel 11427, Pinhais, Paraná, Brazil; 4 Secretaria Municipal de Assistência Social, Prefeitura de Pinhais, Rodovia Deputado João Leopoldo Jacomel 12050, Pinhais, Paraná, Brazil

**Keywords:** animal maltreatment, animal welfare, cat, dog, interdisciplinary actions, One Welfare

## Abstract

A One Welfare approach allows intervention to resolve problems related to the human-animal-environment interface. However, in Brazil and many other countries, there is poor communication between human and animal welfare services. In this research we considered a One Welfare approach in cases of abuse involving dogs and cats. When investigating pet abuse, professionals from the Animal Welfare Division of Pinhais, Brazil, can enter the home environment. During routine animal abuse investigations, the demographic profile of pet owners and their socioeconomic vulnerability was also recorded. Results from forty-five cases with (n = 30) and without (n = 15) suspicion of socioeconomic vulnerability were sent to the Department of Social Assistance of Pinhais, Brazil for confirmation. Socioeconomic vulnerability was suspected if socioeconomic problems were reported by the pet owners under investigation or their neighbours, as well by assessment of the socioeconomic environment of the families during home visits. Economic disadvantage was the most prevalent socioeconomic vulnerability. Cohen’s Kappa coefficients support the ability of animal welfare service professionals to detect socioeconomic vulnerability. Almost half of all families considered to have socioeconomic vulnerability had not previously participated in social programmes. In all cases involving families already being assisted by the Department of Social Assistance, pets were found to be suffering abuse. Families with socioeconomic vulnerability confirmed were included in the social programmes. These results support the need for a multi-disciplinary approach to improve the well-being of families with dogs and cats. This study can help guide the development of an interdisciplinary approach to address animal abuse cases.

## Introduction

A One Welfare approach is necessary to address problems related to the human-animal-environment interface (Segredo 2020). The concept of One Welfare recognises the interconnections between animal welfare, human well-being, and the environment (Pinillos *et al*. 2015). Owners and their animals share some of the same social risks, which affect the welfare of all species involved (Boat & Knight 2001; Degue & Dilillo 2009). Thus, poor human well-being commonly coexists with inadequate animal welfare (Jordan & Lem 2014; Monsalve *et al*. 2018; Shih *et al*. 2019) and animal maltreatment often indicates a human welfare problem (Monsalve *et al*. 2018; Shih *et al*. 2019; Mota-Rojas *et al*. 2022). In this context, the connection between animal abuse and family violence demonstrates the importance of addressing the needs of both human and animal victims and developing a multidisciplinary approach (Peak *et al*. 2012; Jegatheesan *et al*. 2020).

The One Welfare concept requires the interdisciplinary collaboration of different professionals (Jordan & Lem 2014; Pinillos *et al*. 2015; Segredo 2020) and highlights the benefits of animal protection in reducing human and animal suffering (Pinillos *et al*. 2015; Jegatheesan *et al*. 2020). Animal welfare specialists recognise that underlying causes such as community and family dysfunction and violence must be addressed if animal welfare problems are to be resolved (Hoy-Gerlach *et al*. 2019). Additionally, animal welfare professionals and veterinarians have a responsibility to protect, not only the health and safety of the animals under their care, but arguably also the health and safety of the human population (Reese & Ye 2017). Thus, these professionals have a pivotal role in the interdisciplinary approach to social challenges (Colonius & Earley 2013). In this way, an interdisciplinary approach to social problems is essential to protect human and animal well-being. However, poor communication exists between human and animal welfare services in Brazil and other countries (Newberry 2017; Hoy-Gerlach *et al*. 2019; Wuerch *et al*. 2021). Animal health and welfare professionals have traditionally worked independently of the health and social care professions (Jegatheesan *et al*. 2020). Additionally, in most countries, human health services rarely consider the human-animal relationships in their interventions (Peak *et al*. 2012; Newberry 2017; Hoy-Gerlach *et al*. 2019; Wuerch *et al.* 2021).

Brazil has a social policy that seeks to help socioeconomically vulnerable people and families. Socioeconomic vulnerability can be defined as potential loss to a social group arising from a hazard (Venkatesan & Ahmed 2017). It is a complex and multidimensional concept that includes several dimensions such as material, socio-demographic, environmental and affective-relational (Food and Agriculture Organisation 2003; Semzezem & Alves 2013). The policy in Brazil does not set out a definition of socioeconomic vulnerability, but includes conditions related to loss or fragility of family bonds, economic disadvantages, psychoactive substance abuse, violence, and any situation that infringes on fundamental human rights (Ministério do Desenvolvimento Social e Combate à Fome 2004). The Brazilian social policy seeks to improve people’s well-being in many ways including: facilitating access to health, education, and economic programmes, through interdisciplinary actions (Ministério do Desenvolvimento Social e Combate à Fome 2004). However, this policy does not include companion animals or involve animal welfare professionals.

On the other hand, in Brazil, animal abuse is a crime that includes acts of physical, emotional, sexual abuse, neglect, abandonment, and veterinary medical malpractice (CFMV 2018). The sanctions consist of fines and prison from three months to a year for most vertebrate animals (Congresso Nacional do Brasil 1998). In cases involving dogs and cats, prison sentences are 2 to 5 years (Congresso Nacional do Brasil 2020). The municipalities in Brazil have administrative institutions that receive reports of animal abuse. These institutions include veterinarians who carry out animal abuse investigations and decide on the severity of the case. In minor negligence cases, the owner is allowed to improve the conditions of the animals. In severe cases or if the owner does not improve the animal welfare, the animal can be confiscated, and the case sanctioned administratively and criminally. The police can also intervene in cases of animal abuse and request the support of veterinarians. In cases of animal abuse, veterinarians work independently of the health and social care professions. These professionals do not routinely refer their suspicions of human welfare problems to social services, and the socioeconomic vulnerability of the owners of the animals is not addressed.

The aim of this study was to create a One Welfare approach in cases of abuse involving dogs and cats, describing the factors suggesting socioeconomic vulnerability that the animal abuse investigators could detect during home visits, and considering the role of animal welfare professionals in cases of socioeconomic vulnerability.

## Materials and methods

The study was conducted at the Animal Welfare Division of the city of Pinhais, Brazil between April and December 2016. Here, investigations into animal abuse are instigated by a complaint from any member of the public. All animal abuse investigators responsible for verifying complaints of animal abuse are veterinarians. In Pinhais, the legal definition of animal abuse includes non-accidental injuries and intentional or unintentional lack of care (Câmara Municipal de Pinhais 2012). Data were collected during animal abuse investigations that involved owned dogs and cats. During an investigation, home visits are carried out to identify animal neglect or physical aggression within the household. Animal investigators were able to assess the environment of the pet and the owner on these visits.

The research was conducted with the collaboration of the Department of Social Assistance of the same town. This institution co-ordinates social services and identifies and maintains records of socioeconomic vulnerability within families in the municipality. Meetings between professionals of the Animal Welfare Division, the Department of Social Assistance and researchers were held to allow all the institutions to work together.

### Ethical approval

Ethical approval of this study was granted by the Human Research Ethics Committee of the Federal University of Paraná (SCS/UFPR) (Protocol Number 1.502.241). We required ethical permission however since we did not use animals and only collected data about the concepts of animal abuse investigations carried out by the Animal Welfare Division without carrying out any sort of intervention, the Animal Research Ethics Committee of the Federal University of Paraná considered it unnecessary to evaluate this study.

### Determination of the occurrence of animal abuse

In all reports of dog and cat abuse related to lack of care or non-accidental injuries, the Animal Welfare Division of Pinhais carried out a home visit to assess animal welfare. Animal abuse investigators followed the standard protocol for an expert report on animal welfare in cases of suspected companion animal cruelty (Hammerschmidt & Molento 2014). This provides a score for each of the four welfare principles (nutrition, comfort, health, and behaviour) using a combination of both mental state and physical parameters whilst also considering the environmental conditions provided to the pet (Hammerschmidt & Molento 2014). The final score is collated on a five-point scale: very high (all welfare principles adequate), high (one welfare principle regular, other principles classified as adequate), regular (two or more welfare principles regular, other principles classified as adequate), low (one or two welfare principles classified as inadequate) and very low (three or four welfare principles considered inadequate). Both ‘low’ and ‘very low’ are deemed compatible with animal neglect (Hammerschmidt & Molento 2014). During this study, none of the complaints about intentional physical aggression could be confirmed since animals either had no visible injuries, or these could not be evaluated. However, a case was considered ‘suspicious of physical animal abuse’ when a report of intentional aggression was made by a person who lived in the same home as the pet, or by the neighbours.

### Identification of socioeconomic vulnerability

In addition to establishing the occurrence of alleged animal abuse, the investigators recorded information about the socioeconomic profile of the owners of the dogs and cats under investigation. The data collected were: number of residents in the household; gender, age, educational qualifications of residents; and employment or retirement status of those aged over 18 years of age. Specific questions regarding socioeconomic vulnerability were not asked, but the investigators recorded any reports of socioeconomic problems from the pet owners or their neighbours. They also made note of any factors arousing suspicion of socioeconomic vulnerability during the home visits (although there was no pre-prepared list of possible factors that could contribute to a suspicion of socioeconomic vulnerability). All reports of socioeconomic problems and those identified during home visits were used by the animal abuse investigators to determine the common indicators associated with socioeconomic vulnerability (Table 1).

**Table 1.** Factors related to socioeconomic vulnerability identified during home visits by animal abuse investigators of the Animal Welfare Division of Pinhais city

Type of socioeconomic vulnerability	Indicator	Frequency*	
		N	%
Economic disadvantage	Disorganisation and neglect in building maintenance	11	25
	Accumulation of debris	13	30
	Unhealthy environment	13	30
	Report of impossibility to build physical barrier in the house	10	23
	Unemployment reported in half or more adults	14	32
	Report of having difficulties financially supporting the family and animals	15	34
	Report of family being beneficiary of social assistance programmes	9	20
Violence	Child (< 12 years old) alone at home	1	2
	Person with considerable physical or mental disability alone at home	2	4
	Elderly in unhealthy housing with evident lack of self-care	1	2
	Evidence of fear in the woman during inspection in response to the accused man’s reaction	3	7
	Aggressive or intimidating behaviour of the accused	3	7
	Report of the existence of violence	9	20
	Clear lack of care provided to the children	1	2
	Suspected intentional aggression to animals within the family environment	2	4
Substance abuse	Accumulation of containers of alcoholic beverage in the residence	1	2
	Visualisation of a person under the effects of alcohol on more than one occasion	1	2
	Reported of alcohol or drug abuse	3	7
Fragility of family bonds	Report of absence of family bonds or family disagreements	11	25

* More than one factor related to socioeconomic vulnerability could be identified in one home visit, resulting in a cumulative percentage higher than 100%.

The frequencies of the factors related to socioeconomic vulnerability were obtained in a total of 44 cases.

For this study, only one animal abuse investigator assessed all records, classifying each case as ‘socioeconomic vulnerability suspected’ or ‘no socioeconomic vulnerability suspected.’ A classification of suspected socioeconomic vulnerability was made when at least one factor related to one or more type(s) of socioeconomic vulnerability was identified during home visits. The cases where socioeconomic vulnerability was suspected were categorised as:

- Economic disadvantage: families where there were difficulties providing economic support for family members (Ministério do Desenvolvimento Social e Combate à Fome 2012).
- Violence: the intentional use of physical force or power, threatened or actual, against oneself, another person, or against a group or community, that either resulted in (or had a high likelihood of resulting in): injury, death, psychological harm, maldevelopment, or deprivation (World Health Organisation 1996). Intimate partner violence against women, children, elders, abuse of disabled members and self-neglect were included in this group.
- Substance abuse: drug or alcohol abuse by one of the family members.
- Fragility of family bonds: breakdown of emotional and relationship bonds in the family that resulted in the non-protection of its members (Gomes & Pereira 2005; Ministério do Desenvolvimento Social e Combate à Fome 2012). This category included situations where there was absence of emotional support or multiple disagreements between close relatives.

### Confirmation of cases where socioeconomic vulnerability was suspected

Home visits or Department of Social Assistance records were used to confirm socioeconomic vulnerability in pet owners under investigation for animal abuse. Economic constraints lead the Department of Social Assistance to stipulate a limit of 30 cases in which socioeconomic vulnerability would be confirmed. For this reason, the following protocol for referrals was established: cases with known violence or substance abuse were prioritised, as violence causes extreme suffering for the victims and studies have shown a connection between animal abuse and interpersonal violence (Monsalve *et al*. 2017) and substance abuse is considered a factor associated with violence (Fals-Stewart & Kennedy 2005). Households with economic disadvantage were mainly referred to Social Assistance when there was environmental evidence of financial difficulty, or the majority of adult family members were unemployed. Cases considered to have fragile family bonds without another type of socioeconomic vulnerability were referred when the animal owners had no support from any relative.

The Department of Social Assistance verified the cases and sent a report detailing the situation to the Animal Welfare Division. Confirmation of socioeconomic vulnerability was made by social professionals according to the social assistance protocols, based on an interview with pet owners during a home visit or via pre-existing records from referrals. Using the Department of Social Assistance report, all cases with suspected socioeconomic vulnerability were classified as:

- Confirmed cases with socioeconomic vulnerability: when the Department of Assistance Social confirmed the presence of socioeconomic vulnerability.
- Confirmed cases without socioeconomic vulnerability: when the Department of Assistance Social rejected the presence of socioeconomic vulnerability.
- Inconclusive cases: when the Department of Assistance Social was unable to provide information about socioeconomic vulnerability.

### Confirmation of cases where socioeconomic vulnerability was not suspected

To determine the feasibility of detecting socioeconomic vulnerability during animal abuse investigations, cases of animal abuse (very low animal welfare degree) in which socioeconomic vulnerability was not suspected by the animal abuse investigators were also referred to the Department of Social Assistance. The absence of socioeconomic vulnerability was confirmed when animals’ owners were not registered in any programme of the Department of Social Assistance. Here, unlike cases where socioeconomic vulnerability was suspected, home visits could not be carried out due to a lack of resources in the Department of Social Assistance. Fifteen cases with no suspected socioeconomic vulnerability were referred to the Department of Social Assistance. These cases were classified as either confirmed cases with or confirmed cases without socioeconomic vulnerability.

### Data analysis

The factors that raised suspicion of socioeconomic vulnerability for animal abuse investigators were classified according to the type of socioeconomic vulnerability. Additionally, they were further classified according to whether the suspicion of socioeconomic vulnerability was generated by reports from pet owners under investigation, their neighbours, or by direct visualisation of environmental conditions or the family context. Chi-squared Fisher or Chi-square tests were used to determine an association between the reason for suspicion (report or visualisation) and type of socioeconomic vulnerability. The inter-rater agreement between the cases with and without suspicion of socioeconomic vulnerability by the Animal Welfare Division, with their respective confirmation by the Department of Social Assistance, was analysed using Cohen’s Kappa coefficient. Analyses were performed using the IBM SPSS Statistics 19 programme.

## Results

### Identification of cases with socioeconomic vulnerability

Animal abuse investigators from the Animal Welfare Division evaluated socioeconomic vulnerability during investigation of 118 cases of suspected abuse in dogs and cats. The indicators that contributed to the suspicion of vulnerability by animal abuse investigators are listed in Table 1. Of the 118 cases analysed, 90 (76%) were categorised as animal neglect, with a low (n = 48; 41%) and very low (n = 42; 36%) welfare score. Six (5%) cases in dogs were considered ‘suspicious of physical animal abuse’ (Monsalve *et al*. 2018).

There was suspicion of socioeconomic vulnerability in 44 investigations. In 40 (91%) of the cases, the animals were considered neglected, 14 (35%) with low welfare score and 26 (65%) cases with a very low score. In five (13%) cases there was suspicion of physical aggression towards the pet. The socioeconomic vulnerability most often detected was economic disadvantage. In 13 cases (39%) of the 33 families with financial problems, another type of socioeconomic vulnerability was also suspected. The reports of socioeconomic vulnerability by pet owners under investigation for animal abuse and their neighbours were fundamentally important for suspicion of socioeconomic vulnerability. Table 2 shows the classification of cases with suspected socioeconomic vulnerability according to the type and reasons for suspicion.

**Table 2.** Distribution of cases where animal abuse investigators of the Animal Welfare Division of Pinhais were suspicious of socioeconomic vulnerability, classified by type of socioeconomic vulnerability and reason for suspicion

	Total cases with suspicion of socioeconomic vulnerability		Reasons for suspecting socioeconomic vulnerability			
			Report*		Visualisation of the environment/family context*	
Type of socioeconomic vulnerability	N	%	N	%	N	%
Economic disadvantage	33	75	32	73	22	50
Violence	13	30	9	20	9	20
Substance abuse	4	9	3	7	2	4
Fragility of family bonds	11	25	11	25	0	0
Total	44	100	41	93	28	64

* One family could have more than one type of socioeconomic vulnerability, resulting in a cumulative percentage higher than 100%.

The frequencies of the types of socioeconomic vulnerability were obtained in a total of 44 cases.

Pet owners reported economic disadvantages in 32 (73%) of the 44 cases with suspected vulnerability. In 20 (45%) cases with suspicion of socioeconomic vulnerability, people reported different problems relating to their financial situation. Neighbours of the pet owners provided information about their socioeconomic vulnerability during animal abuse investigation in two (5%) of the 44 cases. One of these cases was a report of child neglect and another of substance abuse. When a comparison was made between groups ‘cases with suspicion of economic disadvantage’ (n = 33) and ‘cases with suspicion of another type of socioeconomic vulnerability’ (n = 24), pet owners were more likely to self-report financial difficulties (n = 32; 97%) than other types of problems (n = 18; 75%) in the family (*P* = 0.034).

Home visits allowed the animal abuse investigators to assess the family environment. Thus, in 28 (64%) cases, veterinarians observed some factor that raised the suspicion of socioeconomic vulnerability during animal abuse investigations (Table 2). The most frequently visualised factors were related to economic disadvantage, referring to the sanitary environment and house infrastructure.

There was no statistically significant difference between the number of times that animal abuse investigators identified an observational factor related to the economic context (n = 22; 67%) and the number of times that this professional recorded another type of socioeconomic vulnerability (n = 11; 46%). The suspicion of fragile family bonds was based solely on the reports. In nine (20%) and two (5%) of the cases with suspected socioeconomic vulnerability (n = 44), assessment of the family environment contributed to a suspicion of violence or substance abuse, respectively.

### Confirmation of suspected socioeconomic vulnerability

Thirty cases with suspected socioeconomic vulnerability were referred to the Department of Social Assistance. During home visits in four (13%) of these cases, the social workers rejected socioeconomic vulnerability, three related to economic disadvantages, and one to violence. In two (7%) of the referrals, social worker reports were inconclusive. Thus, in 24 (80%) cases, socioeconomic vulnerability was confirmed. Families in which the occurrence of socioeconomic vulnerability was confirmed were included in the social programmes by the Department of Social Assistance. In six (20%) cases where socioeconomic vulnerability was suspected, the social worker identified other socioeconomic problems not detected by the animal abuse investigators. In most cases with confirmed socioeconomic vulnerability (n = 23; 96%), the animals were abused, all of them victims of neglect and, in four (17%) cases, there was also a suspicion of physical abuse. In all confirmed cases of interpersonal violence (n = 10) and substance abuse (n = 1), the animals were confirmed to be suffering abuse. In 19 (95%) cases with confirmed economic disadvantage and eight (89%) of the confirmed cases with fragile family bonds, animal welfare professionals confirmed animal abuse. In the cases with no suspicion of socioeconomic vulnerability, but with confirmed animal abuse (14 due to animal neglect and one with animal neglect and suspicion of physical aggression) there were no reports of pet owner participation in social programmes in the records of the Department of Social Assistance.

Table 3 shows Cohen’s Kappa coefficients of the agreement between the cases with and without suspicion of socioeconomic vulnerability and cases with and without socioeconomic vulnerability confirmed by Social Assistance. Inter-rater reliability between the cases with and without suspicion of socio-economic vulnerability suspicion with their respective confirmation were above 60% in the four categories of socioeconomic vulnerability (economic disadvantage, violence, substances abuse, and fragility of family bonds). In the economic disadvantage category, the rate was > 80%, showing an almost perfect agreement. Substance abuse had a perfect agreement.

**Table 3.** Distribution of the agreement between the referred cases with and without suspicion of socioeconomic vulnerability by animal abuse investigators and cases confirmed with and without socioeconomic vulnerability by the Department of Social Assistance of Pinhais city, and the respective values of the Cohen’s kappa coefficient

Type of Socioeconomic vulnerability	Institution*				Agreement between institutions						
	Animal Welfare Division		Department of Social Assistance^a^		Agreement		Non-agreement		Inconclusive^b^		
	N	%	N	%	N	%	N	%	N	%	Kappa value
Economic disadvantage											0.81
Cases with	23	51	20	45	19	83	3	13	1	4	
Cases without	22	49	24	53	21	96	1	4	0	0	
Violence											0.79
Cases with	12	27	10	22	8	67	1	8	3	25	
Cases without	33	73	31	69	30	91	2	6	1	3	
Substance abuse											1
Cases with	3	7	1	2	1	33	0	0	2	67	
Cases without	42	93	41	91	41	98	0	0	1	2	
Fragility of family bonds											0.69
Cases with	8	18	9	20	6	75	1	13	1	13	
Cases without	37	82	35	78	34	92	3	8	0	0	
General Socioeconomic vulnerability											0.81
Cases with	30	67	24	53	24	80	4	13	2	7	
Cases without	15	33	19	42	15	100	0	0	0	0	

* The frequencies of the types of socioeconomic vulnerability by institution were obtained in a total value of the 45 referred cases (30 and 15 cases with and suspicion of socioeconomic vulnerability, respectively).

a The missing cases to complete 100% are classified as inconclusive.

b When social assistance could not confirm nor reject the socioeconomic vulnerability in the referred case.

### Benefits for people and animals with socioeconomic vulnerability

Almost half (n = 10; 42%) of the 24 cases with confirmed socioeconomic vulnerability had not received any previous aid from the Department of Social Assistance, nine (90%) of these families had the opportunity to access social programmes. In all families (n = 14) that had previously accessed the social programmes, companion animal abuse was occurring, animal neglect was identified in all cases and in two families physical aggression was also suspected. The Animal Welfare Division subsequently created a programme to help neglected animals in families with socioeconomic vulnerability, before sanctioning the owners. To take part in this programme the owners had to demonstrate an interest in improving the welfare of their pets. Dogs and cats in families with suspected socioeconomic vulnerability were treated as a priority in the public spay/neuter programme and for adoption programmes in cases where the family could not continue with the animal ownership.

## Discussion

Animal health and welfare professionals have traditionally worked independently of the other health and social care professionals (Jegatheesan *et al*. 2020). The One Welfare approach highlights the importance of collaboration between animal welfare professionals, including veterinarians, social workers and human healthcare providers, to improve the health and welfare of people and animals (Jordan & Lem 2014; Pinillos *et al*. 2015).

Animal welfare services include aspects of health services, public safety, social services, policy input, and litigation in animal abuse cases (Reese & Ye 2017). In these ways animal abuse investigators have contact with pet owners and the opportunity to assess the socioeconomic circumstances of owners. Socioeconomic inequalities have a negative effect on quality of life and their identification is essential to help families with socioeconomic vulnerability. In a number of US states any employee of animal welfare services, including veterinarians, must report to social services agencies when they suspect human abuse within the scope of his or her employment (American Veterinary Medical Association 2018). However, to the authors’ knowledge, the participation of animal welfare services in the identification of socioeconomic problems has been little-studied.

Home visits offer professionals from different areas a unique opportunity to detect several risk factors that affect human and animal welfare (Abbey 2009; Drulla *et al*. 2009; Chung *et al*. 2016). In the present study, the home visits for animal abuse investigations allowed animal services’ workers to see the home environment provided to the dogs and cats. They were also able to assess the socioeconomic context of the households by inclusion of demographic questions about pet owners such as: the school attendance of children and adolescents, the economic status of the family, and the presence of a person with physical or mental disabilities within the household. In many cases family members reported some situations that indicated socioeconomic vulnerability. Questions regarding the family profile (to aid understanding of the home environment of the pet) allowed animal owners the opportunity to share their socioeconomic difficulties with professionals. Conversing about animals is a well-known ice-breaker enabling owners’ social problems to be uncovered (Arkow 2016). Thus, an indirect approach, based on information gathered by animal welfare investigators, can contribute to the identification of socioeconomic problems and referrals to the appropriate social services agency. Studies show that people feel more comfortable talking about their socioeconomic problems when questioned indirectly (McCord-Duncan *et al*. 2006; Chung *et al*. 2016). It is also important that professionals demonstrate an interest in solving family problems (McCord-Duncan *et al*. 2006; Chung *et al*. 2016). Specifically, in cases of domestic violence, women feel more comfortable discussing such crimes with health professionals through general inquiries about their relationship with their intimate partner rather than being confronted directly on the subject of potential abuse within the relationship (McCord-Duncan *et al*. 2006).

Animal owners were more likely to report economic disadvantages, possibly because this type of socioeconomic vulnerability can affect the care given to the dogs and cats. Thus, financial problems were commonly used as justification for neglect of a pet. Additionally, in almost all cases with reports of economic disadvantage, animal abuse was confirmed. Studies on companion animal abuse have shown low-income families to have greater difficulty providing basic care for their pets (Carter & Taylor 2017; Monsalve *et al*. 2018; Shih *et al*. 2019). Here, approximately one-third of families with suspected economic disadvantage also had another type of socioeconomic vulnerability. Research has shown that economic difficulty is a risk factor for the occurrence of other types of socioeconomic vulnerabilities such as violence and substance abuse (Slack *et al*. 2011; Junior *et al*. 2015; Chung *et al*. 2016; Yang & Maguire-Jack 2016). Economic disadvantage was the most common self-reported problem, but it was not uncommon for other social problems to be reported to animal abuse investigators. In two home visits, neighbours approached the investigators to provide information about the animal owners’ social condition. This suggests that pet owners and their neighbours may consider animal welfare officials as professionals able to help with the socioeconomic problems of families.

Although the victim’s report is the most accurate way to identify some types of socioeconomic vulnerability such as violence (Cooper *et al*. 2008), fear of retaliation and shame are impediments to vulnerable groups sharing their problems or making a complaint (Fogarty *et al*. 2002; Wanderbroocke & Moré 2013). Thus, the use of indicators based on the assessment of the environmental and family context can be essential to identify socioeconomic vulnerability. In this study, animal abuse investigators reported a number of observational factors that led them to suspect socioeconomic vulnerability, including human violence and substance abuse. Future research should consider these factors and validate their use in home assessment.

Cohen’s Kappa coefficients showed substantial agreement or almost perfect agreement between the suspicion of socioeconomic vulnerability by animal abuse investigators and its confirmation by social workers. Thus, the suspicion of economic disadvantage, violence, and fragility of family bonds by animal welfare professionals was mostly verified by social workers. This shows that animal welfare professionals are able to identify socioeconomic vulnerability during home visits in animal abuse investigations.

A considerable number of families with socioeconomic vulnerability were not in receipt of assistance from social services. In these cases, the detection of socioeconomic vulnerability by animal abuse investigators aided access to social programmes. Communities with financial problems often experience difficulty obtaining government services (Kohl *et al*. 2005). The early identification of socioeconomic vulnerability is fundamental in reducing the consequences to the individuals involved in cases of domestic violence. Thus, for example, the inclusion of violent families in social programmes has been shown to be linked to a decrease in fatal child maltreatment (Douglas & Mohn 2014). Socioeconomic problems are multi-dimensional and require appropriate intervention with the collaboration of government institutions, academia, and other sectors of society (Jones & Logan-Greene 2016). However, veterinarians and other animal welfare professionals have rarely been included in intersectoral actions to address social problems. For example, despite the relationship between animal abuse and domestic violence, agencies that protect people and animals work in a disjointed manner (Peak *et al*. 2012; Long & Kulkarni 2013). The results of this study indicate that animal welfare professionals can detect socioeconomic vulnerability and should be included in intersectoral actions to address social issues.

In most cases with confirmed socioeconomic vulnerability, pets were being abused, including in families previously visited by the Department of Social Assistance. Nevertheless, social workers within this institution do not routinely ask their clients about issues relating to animal welfare. The results of this study show it to be fundamental to include questions about companion animals within social assistance services to reduce animal and human suffering (Peak *et al*. 2012; Hoy-Gerlach *et al*. 2019). The concept of ‘Multispecies households’ accepts that people can openly include their pets as members of the family and recognise that pets can influence some decisions made by their owners, e.g. whether to accept a new job (Faraco & Seminotti 2004; Irvine & Cilia 2017). Some adults delay going into hospital or to refuges because they have no one to provide care for their pet and neglect of owners and their pets frequently coexist (Boat & Knight 2001). Boat and Knight (2001) emphasise the importance of approaching issues with pets within adult protection service programmes because the human-animal bond can influence the inclusion of people in social programmes.

When socioeconomic vulnerability is considered in animal abuse investigations inclusion of the families into animal welfare programmes was prioritised, improving human and animal well-being. Where socioeconomic vulnerability was suspected pets were included in spay/neuter programmes to improve animal welfare and decrease certain health risks. These animals were dewormed, vaccinated, and sterilised. Neutering represents a crucial component of population control, has tangible benefits such as eliminating unwanted reproduction in dogs and cats (lowering the incidence of reproductive disorders and reproductive behaviours) and offers convenience for owners (Root Kustritz 2014). Studies reveal that many who relinquished pets would have retained them had they been able to obtain assistance (Dolan *et al*. 2015). However, it is important to emphasise that, as in cases of child abuse (Egry *et al*. 2015), the presence of socioeconomic problems does not exempt animal owners from their responsibility to provide adequate care for their pets. To the authors’ knowledge, there are no studies that show whether assisting families with socioeconomic vulnerability improves the welfare of the animals; future studies should investigate this topic.

Despite the importance of this research, there are a number of limitations which mean the results should be interpreted with caution. Veterinarians are at the forefront of the health and welfare of animals and the public (Colonius & Earley 2013). In this study the animal abuse investigations were undertaken by veterinarians who, unlike in a number of other countries, had no duty of confidentiality in cases of animal abuse and legal actions (Conselho Federal de Medicina Veterinária 2016). The application of the results of this research by other animal welfare services should consider these factors. In Brazil, most institutions responsible for investigating animal abuse have veterinarians mandated to investigate this crime. However, other professionals such as the police can play a role in animal abuse investigations. Future research should study the role of these professionals in identifying socioeconomic vulnerability.

In this study it was not possible to refer all cases with suspected socioeconomic vulnerability to the Department of Social Assistance and priority was given to cases involving violence and substance abuse. Therefore, although this study found animal abuse investigators were able to identify socioeconomic vulnerability, the results should be applied only to cases similar to the referred cases in this study. Additionally, the workload of the Department of Social Assistance did not allow a home visit to be carried out in all the cases referred, which could have affected the accuracy of confirmation of socioeconomic vulnerability. The evaluation of suspicion of socioeconomic vulnerability by animal abuse investigators and confirmation by social assistance professionals did not utilise the same parameters. However, it is important to consider that one of the intentions of this research was to intervene as little as possible in the routine procedures of the participating institutions. This study is important for supporting the development of intersectoral action between animal welfare and social services and in the identification of factors that allow detection of socioeconomic vulnerability. Animal welfare workers reported several factors that led them to suspect socioeconomic vulnerability, however, future studies should evaluate the applicability of these factors in the identification of families with socioeconomic vulnerability.

### Animal welfare implications

Companion animal welfare is related to family and social well-being. Therefore, solving animal welfare issues requires early identification of, and intervention in, socioeconomic problems of families (Douglas & Mohn 2014; Hoy-Gerlach *et al*. 2019). Animal welfare workers often visit the home environment of owners during animal abuse investigations. Despite its importance, few studies have considered the influence of socioeconomic problems of families on animal welfare. Likewise, there is little record of interdisciplinary and intersectoral actions in cases of animal abuse. This research shows that intersectoral actions are important to improve the well-being of multispecies families and the importance of the animal abuse investigator in the identification of socioeconomic vulnerability. This study can guide the development of interdisciplinary actions to address identification of social vulnerability in companion animal abuse cases. Although families with socioeconomic vulnerability were included in social programmes, the influence of social support on the well-being of people and animals was not determined. Further research is necessary to establish programmes aimed at improving human and animal welfare in families with socioeconomic vulnerability.

## Conclusion

It is valuable to identify socioeconomic vulnerability in families to promote animal welfare and human well-being. This study supports the capacity of animal welfare service professionals to detect socioeconomic vulnerability, and to facilitate the inclusion of at-risk people in social programmes. Therefore, animal welfare services should be included in the intersectoral actions addressing socioeconomic issues. The results of this study indicate that a significant percentage of animals belonging to families with previous social assistance were subject to abuse. Therefore, the inclusion of pets in evaluations by the social assistance institutions is important to help reduce the suffering of animals.
